# Biomethane production from sugar beet pulp under cocultivation with *Clostridium cellulovorans* and methanogens

**DOI:** 10.1186/s13568-019-0752-2

**Published:** 2019-02-18

**Authors:** Hisao Tomita, Fumiyoshi Okazaki, Yutaka Tamaru

**Affiliations:** 1 Department of Life Sciences, Graduate School of Bioresources, Mie University, 1577 Kurimamachiya, Tsu, Mie 514-8507 Japan; 2 Department of Bioinformatics, Institute of Advanced Research Center, Mie University, 1577 Kurimamachiya, Tsu, Mie 514-8507 Japan; 3 Research Center of Smart Cell Innovation, Mie University, 1577 Kurimamachiya, Tsu, Mie 514-8507 Japan

**Keywords:** Methanogenesis, Cellulosic biomass degradation, Coculture, Gas metabolism

## Abstract

This study was demonstrated with a coculture fermentation system using sugar beet pulp (SBP) as a carbon source combining the cellulose-degrading bacterium *Clostridium cellulovorans* with microbial flora of methane production (MFMP) for the direct conversion of cellulosic biomass to methane (CH_4_). The MFMP was taken from a commercial methane fermentation plant and extremely complicated. Therefore, the MFMP was analyzed by a next-generation sequencing system and the microbiome was identified and classified based on several computer programs. As a result, *Methanosarcina mazei* (1.34% of total counts) and the other methanogens were found in the MFMP. Interestingly, the simultaneous utilization of hydrogen (H_2_) and carbon dioxide (CO_2_) for methanogenesis was observed in the coculture with Consortium of *C. cellulovorans* with the MFMP (CCeM) including *M. mazei*. Furthermore, the CCeM degraded 87.3% of SBP without any pretreatment and produced 34.0 L of CH_4_ per 1 kg of dry weight of SBP. Thus, a gas metabolic shift in the fermentation pattern of *C. cellulovorans* was observed in the CCeM coculture. These results indicated that degradation of agricultural wastes was able to be carried out simultaneously with CH_4_ production by *C. cellulovorans* and the MFMP.

## Introduction

Although first-generation biofuels are made from cones and sugarcanes to mainly produce bioethanol by using yeast, they are crops and foods without doubt that led accordingly to the food problem. For certain reasons, second-generation biofuels are produced from non-edible biomass such as agricultural wastes and cellulosic substrates (Naik et al. 2010; Schenk et al. 2008). Furthermore, the investigation of third-generation biofuels made from algae has been started (Alam et al. 2015). Thus, considering competition with food supply in response to an increase in global population, it is desirable to overcome first-generation biofuels, proceeding to next-generation biofuels.

Cellulose is comprised of a linear chain of d-glucose monomers and has strong crystalline (Brethauer and Studer 2015). Moreover, cellulosic biomass is composed of cellulose, hemicellulose and lignin, and has rigid and complex structures (Gray et al. 2006). Hemicellulose is heteropolymer such as xylan, glucuronoxylan, arabinoxylan, glucomannan, and xyloglucan. In addition, lignin, phenol compounds, are assembled with cellulose and hemicellulose. Thus, since rigid and complex structures are constructed in cellulosic biomass, it is very difficult to degrade them enzymatically.

About 20% of the world’s sugars is supplied by a root of a sugar beet (*Beta vulgaris* L.), which are cultivated all over the world mostly in Europe, North America and Russia (FAO—agribusiness handbook 2013). Sugar beet pulp is a by-product of the production of sugar from the sugar beet. The extraction of sugar starts with the cleaning of the sugar beet delivered to the factory, after which the sugar beet is sliced up into small strips (pulp) and then mashed by heating with water to a temperature of approximately 70 °C to dissolve sugars from the pulp. Furthermore, the sugar water and the pulp are separated in an extraction tower. Thus, since sugar beet pulp (SBP) is the residue and non-edible biomass, it was the subject of research into a raw material of second-generation biofuels (Bellido et al. 2015; Zheng et al. 2013). Furthermore, SBP is mainly composed of cellulose, arabinan and pectin, has less lignin. Therefore, SBP is a suitable raw material for second-generation biofuels, because a pretreatment process is not necessary to remove lignin (Table 1).

**Table 1:** Component of sugar beet pulp

Component	Weigh (g) per dry matter (100 g)			
	Hadden et al. (1986)		Zheng et al. (2013)	
Ash	3.42	g/100 g	2.51	g/100 g
Proteins	11.42	g/100 g	11.42	g/100 g
Lipids	1.63	g/100 g	–	
Sugars^a^	5.2	g/100 g	–	
Starch	0.99	g/100 g	–	
Lignin	2.38	g/100 g	1.16	g/100 g
Glucan	17.34	g/100 g^b^	22.7	g/100 g
Xylan	1.36	g/100 g^b^	5.14	g/100 g
Galactan	4.88	g/100 g^b^	5.92	g/100 g
Arabinan	16.83	g/100 g^b^	23.73	g/100g
Mannan	1.58	g/100g^b^	1.85	g/100g
Pectin	21.15	g/100g^b^	22.84	g/100g
Others	–		2.73	g/100g

^a^Total value of rest of fructose, glucose, sucrose and fructan

^b^Conversion of values to polysaccharides in the paper

Some species of Clostridia are known to have ability to degrade cellulosic biomass efficiently using a multi-enzyme complex called the cellulosome and secreted non-cellulosomal enzymes (Doi and Kosugi 2004). Among those species, we have been studying on *Clostridium cellulovorans*, which is a mesophilic and anaerobic cellulolytic bacterium (Sleat et al. 1984). *C. cellulovorans* utilizes not only cellulose but also hemicelluloses consisting of xylose, fructose, galactose, and mannose (Koukiekolo et al. 2005; Beukes et al. 2008; Dredge et al. 2011). Whole-genome sequencing of *C. cellulovorans* and the exoproteome profiles revealed 57 cellulosomal protein-encoding genes and 168 secreted-carbohydrase-encoding genes (Tamaru et al. 2010; Matsui et al. 2013). Furthermore, since high ability of degradation on plant cell walls has so far been reported (Tamaru et al. 2002), researches have continued to study on degradation mechanism for cellulosic biomass such as rice straw by *C. cellulovorans* (Nakajima et al. 2017).

Methane fermentation is conventional-generation biofuels, and many researches have been reported in a wide range of study fields (Guo et al. 2015). Since methane production is carried out by the complex microbial flora included methanogens, it was formerly difficult to grasp the whole of the microbial flora. However, it has now become possible to analyze the whole aspect of the microbiome characteristics using the next-generation sequencing system (Spang et al. 2017). It has been reported on coculturing with *C. cellulovorans* and one of the famous methanogens such *Methanosarcina* spp. (Lu et al. 2017). Since *C. cellulovorans* and methanogens were able to grow anaerobically under mesophilic condition, it was possible to cultivate both of them in a single tank and simultaneously with both the degradation of cellulosic biomass and production of methane (CH_4_).

In the present study, we investigated a process for producing CH_4_ and hydrogen (H_2_) via the coculture of *C. cellulovorans* with microbial flora of methane production (MFMP) that called the Consortium of *C. cellulovorans* with MFMP (CCeM) with carbon sources such as SBP and Avicel. First, we analyzed 16 s rRNA sequences in the MFMP by using a next-generation sequencer. Based on the result of identification of the MFMP microbiome, both *C. cellulovorans* and the MFMP monocultures and the CCeM coculture were carried out to evaluate concentrations of sugars, organic acids, and biogas (H_2_ and CH_4_) yield after cultivation.

## Materials and methods

### Materials

SBP was obtained from a sugar factory in Hokkaido, Japan. It was dried up, milled and sieved through 80 mesh. The substrate concentration of SBP and Avicel (Sigma, MO, USA) was 0.5% (w/v) of dry weight.

### Microorganism and culture condition

The medium was partially modified by *Clostridium cellulovorans* medium (Sleat et al. 1984). One litter medium containing 4 g of yeast extract, 1 mg of Resazurin salt, 1 g of l-cysteine-HCl, 5 g of NaHCO_3_, 0.45 g of K_2_HPO_4_, 0.45 g of KH_2_PO_4_, 0.3675 g of NH_4_Cl, 0.9 g of NaCl, 0.1575 g of MgCl_2_∙6H_2_O, 0.12 g of CaCl_2_∙2H_2_O, 0.85 mg of MnCl_2_∙4H_2_O, 0.942 mg of CoCl_2_∙6H_2_O, 5.2 mg of Na_2_EDTA, 1.5 mg of FeCl_2_∙4H_2_O, 0.07 mg of ZnCl_2_, 0.1 mg H_3_BO_3_, 0.017 mg of CuCl_2_∙2H_2_O, 0.024 mg of NiCl_2_∙6H_2_O, 0.036 mg of Na_2_MoO_4_∙2H_2_O, 6.6 mg of FeSO_4_∙7H_2_O, and 0.1 g of *p*-aminobenzoic acid and was adjusted to pH7. *C. cellulovorans* 743B (ATCC 35296) was used and anaerobically cultivated in 0.5% (w/v) cellobiose (Sigma, MO, USA) at 37 °C for 19 h stationary. The MFMP was obtained from methane fermentation digested liquid on January, 2017 at Gifu in Japan. The MFMP was anaerobically cultivated in *Clostridium cellulovorans* medium with 0.5% (w/v) glucose (Wako) and 0.25% (w/v) cellobiose at 37 °C for 19 h stationary.

## 16S rRNA sequencing

Samples were crashed by Shake Master Neo (bms, Tokyo, Japan) and DNA was extracted by Fast DNA spin kit (MP Bio, CA, USA). MiSeq (Illumina, CA, USA) was used for sequencing under the condition of 2 × 300 bp. Qiime as an analyzing software and Greengene as a database were used, and OTU was decided except chimeric genes.

### Measurement of total sugar and reducing sugar concentration

Total sugar concentration was measured by Phenol–sulfuric acid method. Reducing sugar was measured by DNS method, as d-glucose equivalents.

### Data deposition

The sequences reported in this paper has been deposited in the DDBJ database (accession no. DRR160954).

### Gas concentration

Produced gas after the cultivation was recovered by downward displacement of water, the total gas amount was measured by a syringe (Terumo, Tokyo, Japan). The concentration of CH_4_, H_2_ and CO_2_ was measured by a gas chromatograph GC-8A (Shimadzu, Kyoto, Japan) with TCD detector and a column SINCARBON ST (6 m, inner diameter. 3 mm; Shinwa, Kyoto, Japan). The column temperature was at 200 °C. Argon was a carrier gas and set at a flow rate of 50 mL/min. Injection volume of each sample was 5 mL.

### Organic acid concentration

The concentration of organic acids was measured by high-performance liquid chromatography (HPLC) CBM-20A, LC-20AD, CTO-20AC, SPD-20A and DGU-20A_3_ (Shimadzu, Kyoto, Japan) with UV detector and a column KC-811 (300 mm × 2, inner diameter. 8 mm; Showa Denko, Tokyo, Japan). The column temperature was at 60 °C. The method of BTB Post-column was used. Eluent was 2 mM perchloric acid, and the flow rate was 1.0 mL/min. Reagent was 0.2 mM BTB and 15 mM disodiumhydrogenphosphate, and the flow rate was 1.2 mL/min at the wavelength of 445 nm. Injection volume of each sample was 20 μL.

### Cell growth

Cell growth was measured by Lumitester PD-20, LuciPac Pen and ATP eliminating enzyme (Kikkoman Biochemifa, Tokyo, Japan). It is known that integrated intracellular ATP concentration correlates with cell growth (Miyake et al. 2016). Cell growth was estimated by measuring ATP concentration of 0.1 mL of cell culture according to the manufacturer’s instruction and was expresses by Relative Light Unit (RLU) value.

### Statistics

The data were analyzed for statistical significances using Welch’s *t* test. Difference was assessed with two-side test with an α level of 0.05.

## Results

### Degradation of SBP and Avicel with *C. cellulovorans*

**Fig. 1:**
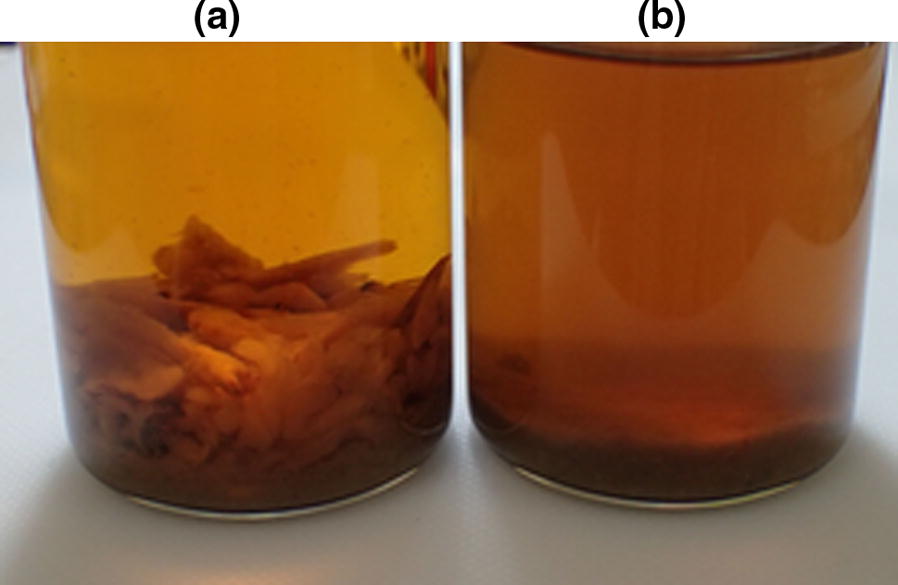
The cultures after the cultivation of *C. cellulovorans* with SBP. **a** Negative control. **b** The cultivation of *C. cellulovorans*. SBP used in the culture media was not pretreated by milling

**Fig. 2:**
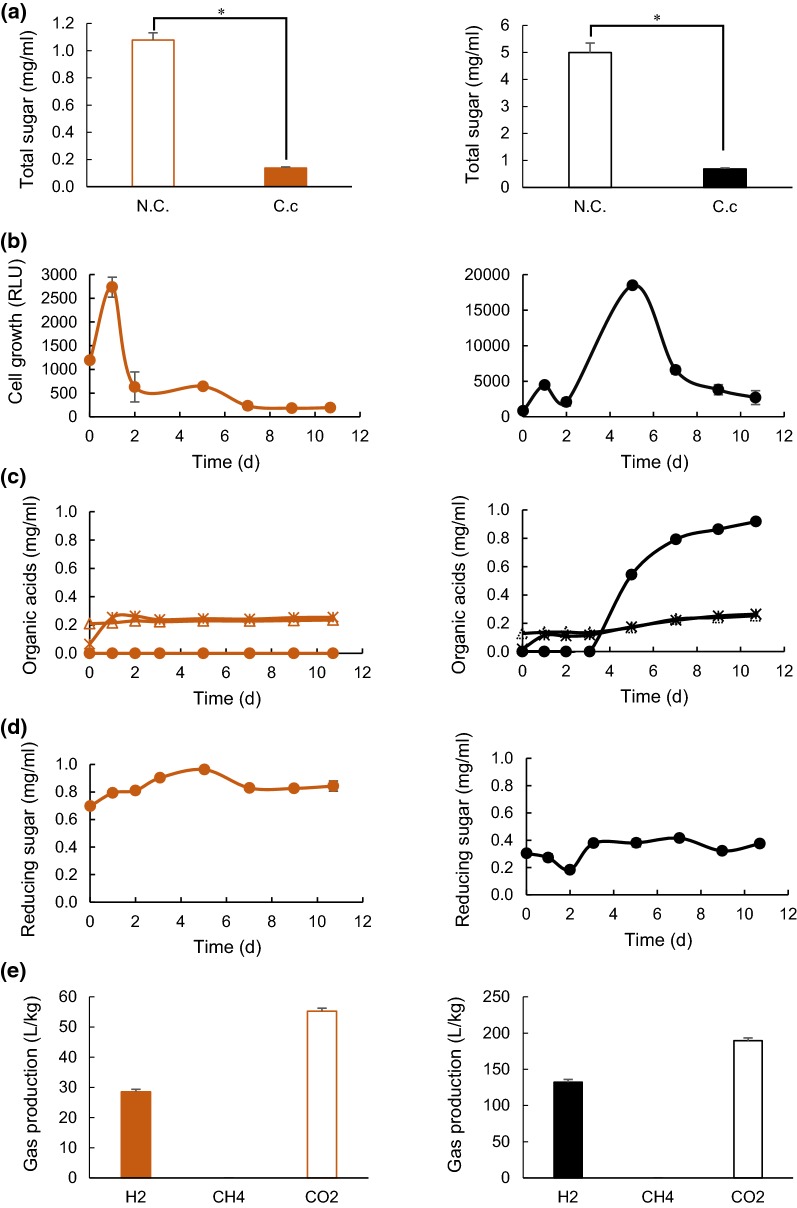
Cultivation of *C. cellulovorans* with SBP and Avicel. **a** Total sugar concentration after 11-days cultivation in the culture with SBP (left) and Avicel (right), where negative control (open bar), *C. cellulovorans* (closed bar) are included. **b** Cell growth in the culture with SBP (left) and Avicel (right). **c** Organic acid concentration in the culture with SBP (left) and Avicel (right), where lactic acid (Δ), acetic acid (*), butyric acid (black filled circle) are included. **d** Reduced sugar concentration in the culture with SBP (left) and Avicel (right). **e** Gas production after 11-days cultivation in the culture with SBP (left) and Avicel (right), where H_2_ (closed bar), CH_4_ (hatched bar), CO_2_ (open bar) are included. Values indicate increments from the volume of negative control and are calculated as the volume per one kg of dry weight of substrates. Values with error bars are mean ± SE of three independent samples. An asterisk indicates a significant difference (p < 0.05)

Anaerobic batch cultivations of *C. cellulovorans* were carried out in a 40-mL medium containing 0.5% (w/v) of SBP at 37 °C without shaking. After cultivation with SBP, the volume became less than half of the negative control (Fig. 1). Next, Avicel was used for a reference of cellulose degradation with *C. cellulovorans*. According to measured cell growth on the precultures, the inoculation volume with a *C. cellulovorans* monoculture was decided. As a result, the initial RLU value of the monoculture closely reached to 1000, whereas the RLU value of the *C. cellulovorans* preculture with 0.5% (w/v) cellobiose was 20,257. Therefore, the inoculation volume was eventually decided to 2 mL for 40-mL monoculture which was about 21 times dilution, so that the initial RLU value of the *C. cellulovorans* monoculture was 964. The concentration of total sugar, reducing sugar and organic acids, cell growth and gas production were measured for 11-days cultivations, respectively. *C. cellulovorans* degraded 87.3% SBP and 86.3% Avicel, respectively, without any pretreatment (Fig. 2a). Interestingly, the profiles of cell growth with *C. cellulovorans* on both SBP and Avicel cultures were completely different (Fig. 2b). The maximum cell growth in the Avicel culture was 5-days after inoculation, while that in the SBP culture was 1-day after inoculation. On the other hand, whereas the concentration of butyric acid increased rapidly on the Avicel culture after 2-days inoculation, there was no increase of butyric acid on the SBP culture (Fig. 2c). It was suggested that a metabolic pathway in *C. cellulovorans* might be different between the SBP and Avicel cultures. According to the concentrations of reducing sugar in the SBP and Avicel cultures, they seemed very similar (Fig. 2d). However, H_2_ productions were 28.6 L per 1 kg of dried SBP and 132 L per 1 kg of Avicel, respectively (Fig. 2e). Therefore, the composition of the reducing sugar in the SBP culture seems reasonable to produce 28.6 L of H_2_ whose concentration was close to 22% of 132 L of H_2_ in the Avicel culture. Thus, it indicated that *C. cellulovorans* degraded cellulosic biomass to produce H_2_ which should be a raw material of CH_4_ by the CO_2_ reduction pathway in methanogens.

### All-inclusive analysis of microbial flora included methanogens

**Fig. 3:**
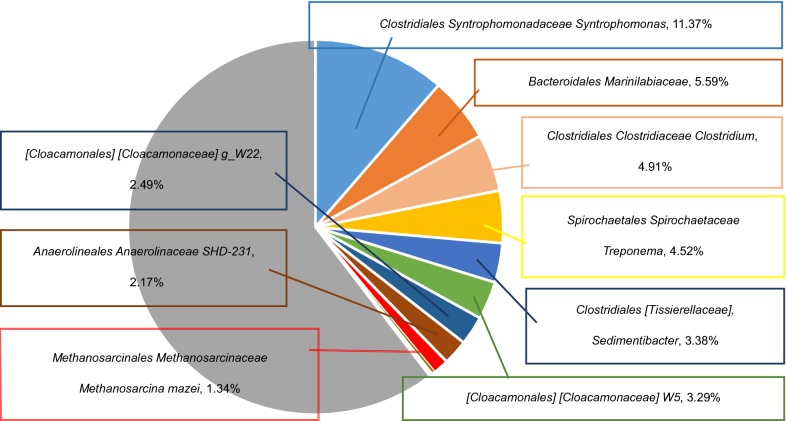
Dominant families in MFMP by 16S rRNA sequencing

Based on the 16S rRNA sequencing, a total of 2359 OUT IDs has read counts from analyzing 24,105 OUT IDs. Eventually, 17 classes and their species were identified among them (Table 2). In fact, whereas *Clostridium butyricum* was identified as the same species of *C. cellulovorans*, *Methanosarcina mazei* (1.34%) was found among methanogens. Furthermore, other methanogens such as *Methanosaetaceae*, *Methanosaeta*, and *Methanospirillaceae* were also identified. More interestingly, the genus *Methanosaeta*, which utilizes only acetic acid, was a large portion of ratio next to *Methanosarcina* (Table 3). Dominant families were identified and belonging to *Syntrophomonadaceae* (11.37%), *Marinilabiaceae* (5.59%), *Clostridiaceae* (4.91%), and *Spirochaetaceae* (4.52%) (Fig. 3).

**Table 2:** Identification of 17 classes and their species by 16S rRNA sequencing

Kingdom	Phylum	Class	Order	Family	Genus	Species	Ratio (%)
*Archaea*	*Euryarchaeota*	*Methanomicrobia*	*Methanosarcinales*	*Methanosarcinaceae*	*Methanosarcina*	*mazei*	1.340
*Bacteria*	*Thermotogae*	*Thermotogae*	*Thermotogales*	*Thermotogaceae*	*Kosmotoga*	*mrcj*	0.278
*Bacteria*	*Firmicutes*	*Clostridia*	*Clostridiales*	*Syntrophomonadaceae*	*Syntrophomonas*	*wolfei*	0.099
*Bacteria*	*Firmicutes*	*Clostridia*	*Clostridiales*	*Peptococcaceae*	*Desulfosporosinus*	*meridiei*	0.073
*Bacteria*	*Fibrobacteres*	*Fibrobacteria*	*Fibrobacterales*	*Fibrobacteraceae*	*Fibrobacter*	*succinogenes*	0.039
*Bacteria*	*Proteobacteria*	*Epsilonproteobacteria*	*Campylobacterales*	*Campylobacteraceae*	*Arcobacter*	*cryaerophilus*	0.009
*Bacteria*	*Firmicutes*	*Clostridia*	*Clostridiales*	*Clostridiaceae*	*Clostridium*	*butyricum*	0.005
*Bacteria*	*Actinobacteria*	*Actinobacteria*	*Actinomycetales*	*Actinomycetaceae*	*Actinomyces*	*europaeus*	0.002
*Bacteria*	*Proteobacteria*	*Gammaproteobacteria*	*Pseudomonadales*	*Moraxellaceae*	*Acinetobacter*	*lwoffii*	0.002
*Bacteria*	*Bacteroidetes*	*Bacteroidia*	*Bacteroidales*	*Bacteroidaceae*	*Bacteroides*	*ovatus*	0.002
*Bacteria*	*Firmicutes*	*Bacilli*	*Bacillales*	*Bacillaceae*	*Bacillus*	*flexus*	0.001
*Bacteria*	*Proteobacteria*	*Gammaproteobacteria*	*Vibrionales*	*Vibrionaceae*	*Vibrio*	*fortis*	0.001
*Bacteria*	*Firmicutes*	*Bacilli*	*Lactobacillales*	*Lactobacillaceae*	*Lactobacillus*	*manihotivorans*	0.001
*Bacteria*	*Proteobacteria*	*Gammaproteobacteria*	*Enterobacteriales*	*Enterobacteriaceae*	*Serratia*	*marcescens*	0.001
*Bacteria*	*Actinobacteria*	*Actinobacteria*	*Actinomycetales*	*Nocardiaceae*	*Rhodococcus*	*ruber*	0.001
*Bacteria*	*Bacteroidetes*	*Flavobacteriia*	*Flavobacteriales*	*Flavobacteriaceae*	*Flavobacterium*	*succinicans*	0.001
*Bacteria*	*Proteobacteria*	*Alphaproteobacteria*	*Rhizobiales*	*Hyphomicrobiaceae*	*Hyphomicrobium*	*sulfonivorans*	0.001

**Table 3:** Identification of methanogens by 16S rRNA sequencing

Kingdom	Phylum	Class	Order	Family	Genus	Species	
*Archaea*	*Euryarchaeota*	*Methanomicrobia*	*Methanosarcinales*	*Methanosarcinaceae*	*Methanosarcina*	*mazei*	1.34%
*Archaea*	*Euryarchaeota*	*Methanomicrobia*	*Methanosarcinales*	*Methanosaetaceae*	*Methanosaeta*		0.54%
*Archaea*	*Euryarchaeota*	*Methanomicrobia*	*Methanomicrobiales*	*Methanospirillaceae*			0.25%
*Archaea*	*Euryarchaeota*	*Methanomicrobia*	*Methanomicrobiales*	*Methanomicrobiaceae*	*Methanoculleus*		0.22%
*Archaea*	*Euryarchaeota*	*Methanomicrobia*	*Methanomicrobiales*	*Methanocorpusculaceae*	*Methanocorpusculum*		0.09%
*Archaea*	*Euryarchaeota*	*Methanomicrobia*	*Methanosarcinales*	*Methanosarcinaceae*	*Methanosarcina*		0.09%
*Archaea*	*Euryarchaeota*	*Methanomicrobia*	*Methanomicrobiales*	*Methanomicrobiaceae*	*Methanofollis*		0.07%
*Archaea*	*Euryarchaeota*	*Methanobacteria*	*Methanobacteriales*	*Methanobacteriaceae*	*Methanobacterium*		0.04%
*Archaea*	*Euryarchaeota*	*Methanobacteria*	*Methanobacteriales*	*Methanobacteriaceae*	*Methanobrevibacter*		0.02%
*Archaea*	*miscellaneous*						0.25%

### Precultivation of *C. cellulovorans* and MFMP

The inoculation volume to the MFMP monoculture was decided as same as the *C. cellulovorans* monoculture, so that the initial RLU values of each monoculture closely reached to 1000. The RLU value of the MFMP preculture with 0.5% (w/v) glucose and 0.25% (w/v) cellobiose was 14,812. Therefore, the inoculation volume was decided to 3 mL for 40-mL monoculture, so that the initial RLU value of the MFMP monoculture was 1036. 2 mL of the *C. cellulovorans* preculture and 3 mL of the MFMP preculture, respectively, were inoculated in the CCeM culture, in order that the concentration of cell growth against the substrate became same as monocultures.

### Methanogenesis and SBP utilization

**Fig. 4:**
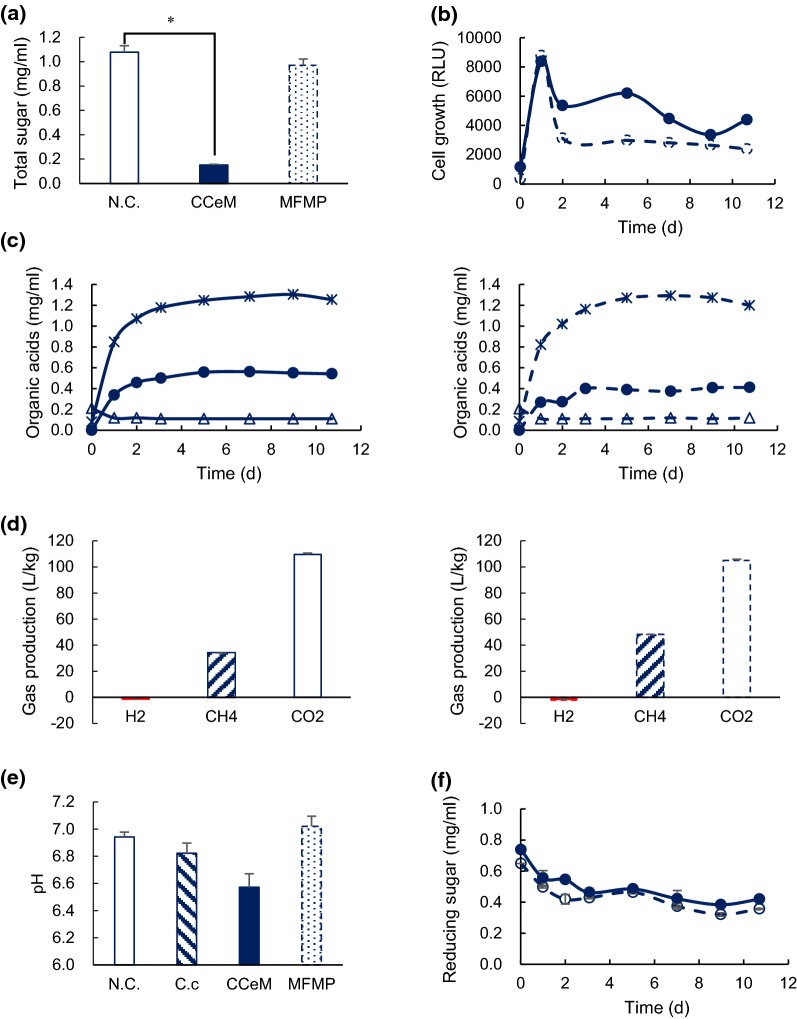
Cultivation of *C. cellulovorans*, CCeM and MFMP with SBP. **a** Total sugar concentration after 11-days cultivation in the culture with SBP, where negative control (open bar), CCeM (closed bar), MFMP (dotted bar) are included. **b** Cell growth in the culture of CCeM and MFMP with SBP, where CCeM (open circle), MFMP (closed circle). **c** Organic acid concentration in the CCeM (left) and MFMP (right) cultures with SBP, where lactic acid (Δ), acetic acid (*), butyric acid (black filled circle) are included. **d** Gas production after 11-days cultivation in the CCeM (left) and MFMP (right) cultures with SBP, where H_2_ (closed bar), CH_4_ (hatched bar), CO_2_ (open bar) are included. Values indicate increments from the volume of negative control and are calculated as the volume per one kg of dry weight of SBP. **e** pH after 11-days cultivation with SBP, where negative control (open bar), *C. cellulovorans* (hatched bar), CCeM (closed bar), MFMP (dotted bar) are included. **f** Concentration of reducing sugar in the CCeM and MFMP cultures with SBP, CCeM (closed circle), MFMP (open circle). Values with error bars are mean ± SE of three independent samples. An asterisk indicates a significant difference (p < 0.05)

Anaerobic batch cultivations of the CCeM and MFMP cultures were carried out in a 40-mL medium containing 0.5% (w/v) of sugar beet pulp at 37 °C without shaking. The total sugar of the MFMP culture was hardly decreased. However, surprisingly, the total sugar of the CCeM culture decreased 86.0% that was not significantly deference compared with that of *C. cellulovorans* monoculture (Fig. 4a). Furthermore, cell growth of the CCeM culture was higher than that of the MFMP culture during 2–6 days cultivation as with the RLU profile of the *C. cellulovorans* monoculture (Fig. 4b), and the butyric acid concentration in the CCeM culture was higher than that in the MFMP culture (Fig. 4c). Whereas pH in the *C. cellulovorans* culture with Avicel after 11-days cultivation was 6.4 due to the high concentration of butyric acid, pH in the CCeM culture with SBP was 6.57 (Fig. 4e). On the other hand, the reducing sugar concentration decreased from the initial value in the CCeM and MFMP cultures, and CO_2_ production in both cultures were two times higher than that in the *C. cellulovorans* monoculture (Fig. 4f). It suggested that various microbes in the MFMP consumed the reducing sugar and produced CO_2_ in the CCeM and MFMP cultures. Thus, it was demonstrated that *C. cellulovorans* was able to coexist with methanogens and various other microbes to degrade SBP, while the degradation performance of *C. cellulovorans* was maintained. For biogas production, 34.0 L/kg of CH_4_ and 110 L/kg of CO_2_ were measured in the CCeM culture, respectively. On the other hand, 48.2 L/kg of CH_4_ and 105 L/kg of CO_2_ in the MFMP culture were done, respectively. It was also revealed that MFMP was able to produce CH_4_ coexisting *C. cellulovorans*. More interestingly, H_2_ was not accumulated in both cultures, and the final volume of H_2_ was less than that in negative control, although 28.6 L/kg H_2_ was produced in the *C. cellulovorans* monoculture (Fig. 4d). These results suggested that *M. mazei* generated CH_4_ from H_2_ and CO_2_ by the CO_2_ reduction pathway.

## Discussion

The biomethanation process is not a single process. Three anaerobic microbes such as fermentative microbes, acetogenic microbes and methanogens mainly participate in the methanation (Hattori 2008; Thauer et al. 2008; Garcia et al. 2000). In fact, methanogens required acetate, H_2_ and CO_2_, which are precursors for methanogenesis, to metabolite CH_4_ by two major pathways such as the acetoclastic pathway and the CO_2_ reduction pathway (Deppenmeier et al. 1996). Fermentative and acetogenic microbes degrade organic matters and supply the precursors to methanogens. A physiological and molecular investigation of two artificially constructed co-cultures with *C. cellulovorans*–*M. barkeri* utilizing cellulose as the sole carbon source has been reported (Lu et al. 2017). In this study, whereas *C. cellulovorans* produced H_2_, acetate, butyrate, and lactate as the obligatory fermentation products from cellulose degradation, *M. barkeri* was able to further utilize H_2_, formate, and acetate for methanogenesis by both the CO_2_ reduction and acetoclastic pathways.

**Fig. 5:**
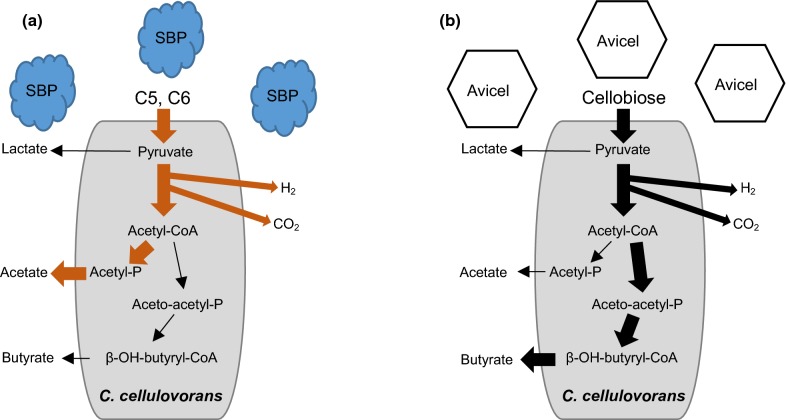
Metabolic pathways in *C. cellulovorans*. The pathway of organic acid production with SBP (**a**) and Avicel (**b**) as a substrate

In this study, we demonstrated that the CCeM was able to degrade SBP and produce CH_4_ simultaneously in a single tank. In fact, SBP included highly suitable substrates for bioconversion by the CCeM. Although *C. cellulovorans* was able to grow on the medium containing 0.5% cellobiose, some bacteria can never utilize it. In fact, after cultivation of *C. cellulovorans* with the Avicel medium, main hydrolyzed products were cellobiose in the supernatant, suggesting that only glucose might be used for methane production by MFMP. On the other hand, since however *C. cellulovorans* degraded SBP to produce a variety of saccharides which could be utilized by various microbes in MFMP. Therefore, SBP would be a great benefit to reduce the cost of drying and transporting SBP in sugar factories. Exoproteome analysis of *C. cellulovorans* under the cultivation with several substrates such as bagasse, corn germ, and rice straw revealed that 18 of the proteins were specifically produced during degradation of types of natural soft biomass (Esaka et al. 2015). More interestingly, in comparison of the cocultures between *C. cellulovorans*–*M. berkeri* and *C. cellulovorans*–*M. mazei*, the pattern of gene expression on a cellulose encoding Clocel_0905 was completely different from the combination between *M. berkeri* and *M. mazei* (Lu et al. 2017). This result indicated that it might have another possibility of cellulose degradation manners via microbial interactions. In this study, the concentration of butyric acid in SBP culture did not increase much, although that of acetic acid immediately increased for 1-day cultivation (Fig. 4c), suggesting that *C. cellulovorans* grew and produced butyric acid and the starting point of its growth was delayed until cellulosome and non-cellulosomal enzymes were secreted and accordingly started to degrade Avicel (Fig. 2b, c). Therefore, it was suggested that a metabolic pathway seems to be different between the SBP and Avicel cultures (Fig. 5).

Shinohara et al. (2013) reported fixation of CO_2_ in *C. cellulovorans* by partly operated the TCA cycle in a reductive manner. In this study, *C. cellulovorans* has been suggested to have a CO_2_ fixation pathway, because of its ability to grow under a higher concentration of 100% CO_2_ compared to other *Clostridium* species. In the genome analysis of *C. cellulovorans* (Tamaru et al. 2010), the genes of two important CO_2_ fixation enzymes, namely pyruvate ferredoxin oxidoreductase (PFOR) and phosphoenolpyruvic acid (PEP) carboxylase (PEPC) were annotated. More interestingly, PFOR of glycolysis and PEPC of the TCA cycle are both in the node of main metabolic pathways in *C. cellulovorans*. At this point, *C. cellulovorans* produced 132 L/kg of H_2_ and 190 L/kg of CO_2_ under the cultivation of Avicel medium. Therefore, if these gases are completely converted to CH_4_ through CO_2_ reduction pathway in methanogens, more H_2_ is theoretically required for CH_4_ production.

Although much is not known of the mechanisms that create and maintain *Methanosarcina* diversity in any given environment, the distinct metabolism of the clade likely has a role (Youngblut et al. 2015). In addition, gene gain from bacterial taxa is common in at least some *Methanosarcina* spp. and may often be adaptive (Deppenmeier et al. 2002; Fournier and Gogarten 2007). Host mobile element dynamics may also have a key role, given that *Methanosarcina* genomes contain a large number of putative mobile element genes and all contain multiple clustered regularly interspaced short palindromic repeats (CRISPRs) (Maeder et al. 2006; Nickel et al. 2013). Based on the 16S rRNA sequencing, *M. mazei* and the other methanogens were found in MFMP (Table 2, Fig. 3). In addition, various other miscellaneous microbes also existed. These results revealed that by using RLU as an index to construct the consortium, *C. cellulovorans* could survive with MFMP by setting the RLU ratio of *C. cellulovorans* and the MFMP that each of the initial RLUs was decided to 1 and 1000, respectively. In terms of CH_4_ yield from SBP, 502.5 L/kg of CH_4_ yields by using hydrothermal pretreatment and 360 L/kg by adding of external enzymes has been reported (Ziemiński et al. 2014; Miroslav et al. 2000). Although 34.0 L/kg of CH_4_ yield in this study was lower than these reports, this study did not require any pretreatments and extra enzymes, suggesting that this study would have much advantages on a cost–benefit. In addition, since the yield depends on the saccharides concentration in SBP, the efficiency of sugar refinery in sugar factories would be able to control CH_4_ yield. In fact, CH_4_ production in the CCeM culture was lower than that in the MFMP culture, from another point of view, the volume reduction of SBP by *C. cellulovorans* is able to compensate the drying and transporting energy (Fig. 1). Furthermore, an adjusting the RLU ratio or pH in the CCeM culture are ways to improve CH_4_ production. More interestingly, since the RLU value in the CCeM was extremely higher than the total value of the RLU value in the SBP monoculture and the MFMP culture (Fig. 2b), *C. cellulovorans* seems to interact with not only methanogens but also miscellaneous microbes. Therefore, there might have some possibilities that growing miscellaneous microbes in the CCeM increase their RLU and inhibit CH_4_ production.

In future study, it could be possible to find various factors that are not gained from the coculture between *C. cellulovorans* and methanogens through omics analysis. Furthermore, by the machine learning using these data (Charlson et al. 2010), there are some possibilities that these omics data are able to elucidate not only inhibit factors for CH_4_ production, but also interrelationship between each microbe in the CCeM.
